# Phytochemical Analysis and Anti-microbial Activity of Some Important Medicinal Plants from North-west of Iran

**DOI:** 10.22037/ijpr.2019.1100817

**Published:** 2019

**Authors:** Samaneh Karimi, Farzaneh Lotfipour, Solmaz Asnaashari, Parina Asgharian, Yaser Sarvari, Saeid Hazrati

**Affiliations:** a *Immunology Research Center, Tabriz University of Medical Sciences, Tabriz, Iran. *; b *Department of Pharmaceutical and Food Control, Faculty of Pharmacy, Tabriz University of Medical Sciences Tabriz, Iran. *; c *Biotechnology Research Center, Tabriz University of Medical Sciences, Tabriz, Iran. *; d *Drug Applied Research Center, Tabriz University of Medical Sciences, Tabriz, Iran. *; e *Department of Pharmacognosy, Faculty of Pharmacy, Tabriz University of Medical Sciences, Tabriz, Iran. *; f *Research Institute for Fundamental Sciences (RIFS), University of Tabriz, Tabriz, Iran. *; g *Department of Agronomy, Faculty of Agriculture, Azarbaijan Shahid Madani University, Tabriz, Iran.*

**Keywords:** Antimicrobial effect, Eryngium caeruleum, Eryngium thyrsoideum, GC-MS analysis, phytochemical profile

## Abstract

Due to the increase of microbial resistance to antibiotics and the occurrence of side effects, use of medicinal plants with anti-microbial properties seems to be rational. Hence, in this study, some plants of the Apiaceae, Asteraceae, Brassicaceae, and Cucurbitaceae families were evaluated for antimicrobial effects. The aerial parts of the plants were extracted by different solvents using a Soxhlet apparatus. Subsequently, the inhibitory effect of the extracts on different microbial species was assessed. Extracts with high growth inhibitory effect were fractionated and their MIC was determined. Furthermore, primary phytochemical and GC-MS analysis were used to identify the chemical compounds of potent samples of n-hexane extracts of *Eryngium caerulum *(*E. caeruleum*) and *Eryngium thyrsoideum *(*E. thyrsoideum*.) Both plants showed considerable antimicrobial activities against *Staphylococcus epidermidis* among the fractions, 40% and 60% VLC fractions of n-hex extract of *E. caeruleum* and 40% VLC fraction of n-hexane extract of *E. thyrsoideum *illustrated the most growth inhibitory effect. Moreover, the results of preliminary phytochemical and GC-MS analysis confirmed that steroids, fatty acids and terpenoids play an important role to show anti-microbial activity, respectively. Among all samples, the 40% VLC fraction of n-hexane extract of *E. thyrsoideum* for possessing high amounts of fatty acids and terpenoids indicated the most anti-microbial potency.

## Introduction

Infectious illnesses are recognized as one of the significant leading sources of morbidity and mortality in the world (1). The anti-microbial compounds which can retard microbial growth or cause microbial mortality with minimum toxicity to host cells are deliberated campaign for promoting anti-microbial substances. Furthermore, due to the growing increase of multiple antibiotic resistance in pathogenic microorganisms in the last two decades from all over the world and also to the development of adverse effects of main antibiotics and the occurrence of previously unusual infectious diseases, a search for new plants with anti-microbial properties to determine new natural antibiotics which might decrease mentioned complications (2-6). Herbal medicines are the main sources of natural drugs which can be a substitute to the common medicines (7, 8). Recently, anti-microbial properties of herbal drugs are being progressively identified in the most parts of the world (9-16). In this research project, the anti-microbial activities of some plants traditionally used by people of Iran, were screened against different microbial species including gram positives, gram negatives, and a fungi. Moreover, preliminary qualitative phytochemical as well as GC-MS analysis of potent extracts and fractions have been illustrated which phytochemicals are responsible for showing antimicrobial effect. The plants, examined in this study, were generally used to cure the infections and related symptoms (Table 1). In this regard, we report the conclusion of this study for future research planning in order to find effective and non-toxic anti-microbial medicines.

## Experimental

*Plant material*

Seven species of 4 authenticated plant families used in the traditional medicine by Iranian people were collected from different regions of Iran. All of the plants, after identification by a senior herbal taxonomist, Dr. Atefeh Ebrahimi, have been deposited at the herbarium of the Faculty of Pharmacy, Tabriz University of Medical Sciences, Tabriz, Iran.

*Preparation of plant extracts and fractions*

Dried powdered aerial parts of *Antriscus nemorosa*, *Artemisia* *marschalliana*, *Eryngium caeruleum*, *Eryngium thyrsoideum*, *Lepidium* *vesicarium* and also the seeds of *Eryngium* *elaterium* were extracted by soxhlet apparatus using n-hexane (n-hex), dichloromethane (DCM), and methanol (MeOH) (1.1 L each, Caledon company, Canada). The filtered extracts were separately dried and concentrated by using a rotary evaporator vacuum at 45 °C. Dried extracts and fractions were diluted in DMSO to prepare the stock concentration 100 mg mL^-1^ for anti-microbial tests.

*Antimicrobial bioassay*

*Test microorganisms*

Anti-microbial activity of plant extracts were tested against four gram positive strains including *Staphylococus aureus* (ATCC 6538), *Staphylococus epidermidis* (ATCC 12228), *Bacillus subtilis* (ATCC 9372), *Listeria monocytogenes *(PTCC 1163), two negative strains *Escherichia coli* (ATCC 8739),* Salmonella typhi* (PTCC 1230), and a fungi *Candida albicans *(ATCC 10231). Lyophilized form of these microorganisms was prepared from Pasture institute, Tehran, Iran.

*Disk diffusion method*

In this method, Muller-Hinton-agar plates were cultured with a standard 0.5 McFarland (10^8^ CFU/mL) inoculum of the test microorganisms. Then 4 filter paper discs (about 6 mm in diameter) and one standard antibiotic disk (positive control) were placed on the surface of each inoculated plate. Forty microliter of each extract solution was transferred over 3 blank disks and one disk was impregnated with DMSO as a negative control. In the next step, they were incubated in 37 °C for 24 h. Finally, for evaluating the anti-microbial activity, the diameter of inhibition zone against the tested organisms was measured by a caliper (16).

*Minimal Inhibitory Concentration (MIC) determination*

For determining the MIC of potent extracts and their fractions, modified broth micro-dilution method was used. In this test, 100 μL distilled water was added to each well of a sterile 96-well microtitre plate and then 100 μL extract solution (100 mg mL^-1^) was added to first well and was diluted two-fold serially until eighth well. Afterwards, 100 μL of each bacterial suspension (5 × 10^5 ^CFU/mL) in Muller Hinton Broth (MHB) medium was added until eight well. Three control wells were filled with 1) DMSO and culture medium containing bacterial suspension, 2) culture medium containing bacterial suspension only and 3) culture medium with plant extract, respectively. The final volume in each well was 200 μL. After well-mixing, the microtitre plate was incubated for 24 h. Finally, the lowest concentration that totally inhibited the growth of microorganism was determined as the MIC and the test was repeated three times (17).

*Fractionation of non-polar extracts*

For further investigations, the potent extracts (inhibited strongly growth of the bacteria) were subjected to Vacuum Liquid Chromatography (VLC) method. VLC is a method to obtain different fractions of non-polar extracts (18, 19). In this method, first VLC hopper was connected to Buchner filter, and then a filter paper was put on the filter. In the next step, the silica gel was loaded into the 2/3 of the tightened hopper. Subsequently, the vacuum pomp was used to compact the silica gel. Another filter paper was placed on the silica gel column. After these preparations, 150 mL methanol, 150 mL ethyl acetate and 150 mL ethyl acetate 10% (10% Et OAc and 90% n-hex) were passed over the silica gel, respectively. Then, the filter paper over the column was removed. Afterwards, n-hex extract was solved in adequate EtOAc 10% and then the solution was loaded over the column and then step by step several concentrations of Et OAc in n-hex (10%, 20%, 40%, 60%, 80% and 100%) were passed through the column and were collected in separate containers. In the end, the pure methanol passed over the silica gel to purify the column.

*Preliminary phytochemical analysis*

*Phytochemical analysis*

*A: Steroids and Triterpenoids tests*

Libermann-Buchard test: little amount of acetic anhydride were added to the different samples and mixed together. Subsequently, by adding the concentrated H_2_SO_4_ slowly, brown ring at the gap of two layers was observed through one hour. The color changes in up and down layer were used to distinguish the steroids from triterpenoids. Formation of bluish green and red color in upper and down layer was considered for the presence of steroids and triterpenoids, respectively (16).

*B: Tests for Glycosides*

*Libermann- Buchard test*

The Libermann- Buchard test as the primary test proved the presence of Steroids and Triterpenoids. Correspondingly, Kedd and keller killiani tests as main characteristic of the cardiac glycoside Components, were applied for determining the existence of unsaturated lactones and deoxy sugars, respectively. 

*Kedd΄s test*

2-3 drops of (3, 5 dinitro benzoic acid) 2%, in 90% alcohol were added to dry samples, then the PH of Kedde΄s reagent with 20% KOH was fixed in alkali range. Formation of purple color indicated the presence of β-unsaturated -O- lactones. 

*Keller-killiani test*

The mixture of CH_3_COOH and FeCl_3_ was added to the dried samples. Following, by adding the concentrated H_2_SO_4_, the color changes were observed to bluish green and reddish in upper and down layers, respectively. It is better to add the sulfuric acid slowly by the flank of the sample tube.

*C: Tests for Alkaloids*

*Dragendorff’s test*

Brownish precipitate was observed by adding the potassium bismuth iodide solution to the samples (Dragendorff reagent). 

*Hagerʹs test*

The test specimens which contain alkaloids show yellow precipitate in presence of Hager reagent (Saturated solution of picric acid). 

*D: Test for Tannins*

Dark green and blue color appeared in the samples which have tannins, by adding the 5% Ferric chloride.

*E: Test for flavonoids*

*Shinoda test*

Amount of HCL 37% with all test samples was mixed, Magnesium ribbon was added for accelarating the speed of color changes to red.

*G: Test for Carbohydrate*

*Benedict’s test*

The blending of the test samples and Benedict’s reagent was boiled in water bath. Subsequently, brownish precipitation in presence carbohydrates was observed (16).

*GC-MS analysis*

GC/MS analysis of the potent extracts and fractions of potent samples was carried out on a Shimadzu GCMS-QP5050A gas chromatograph-mass spectrometer (GC-MS) fitted with a fused methyl silicon DB-1 column (60 m × 0.25 mm i.d., 0.25 μm film thickness). Carrier gas was helium with a flow rate of 1.3. The column temperature was programmed at 100 °C for 2 min and then increased to 300 °C at a rate of 4 °C/min increase, and finally kept constant at 300 °C for 15 min. The injector temperature was 270 °C and split ratio was set up at 1:19. The injection volume was 1 mL. The mass operating parameters were obtained at the following conditions: ionization potential 70 eV; ion source temperature 260 °C; quadrupole temperature 100 °C; solvent delay 2.0 min; resolution 2000 amu/sec, and scan range 30-600 amu; EM voltage 3000 volts.

Components of the potent samples (extracts and fractions) were identified by standard compounds, and computer matching with the NIST 107, NIST 21, NIST 69, and Wiley 229 mass spectral database, as well as by comparison of the fragmentation patterns of the mass spectra with those reported in the literature (16). For quantitation (area%), the GC analysis was also performed using an Agilent 6890 gas chromatograph equipped with a FID detector. The FID detector temperature was 300 ºC.

To obtain the same elution with GC/MS, duplicatation of the same column and same operational conditions was applied. Relative percentage amounts of peak areas were calculated from FID chromatograms.

## Results

The results of disk diffusion method and MIC determination were illustrated in Tables 2 and 3. According to Table 2, the results of screening are promising, among the extracts, 12 extracts illustrated anti-microbial activity against one or more bacteria while 2 extracts (methanolic and n-hexane extracts of *A. nemorosa* and *L. vesicarium *respectively) indicated anti-candida potency. Among the all extracts (Table 2), only the n-hex extracts of *E*. *caeruleum* and *E*. *thyrsoideum* caused significant anti-microbial effects on *S. aureus* and *S. epidermidis*. In the cases of VLC fractions of n-hex extracts (Table 3), 60% and 40% VLC fractions of *E. caerulum* and also 40% VLC fraction in *E. thyrsoideum* against *S. epidermidis* (MIC = 1.562, 1.562 and 0.39 mg mL^-1^, respectively) exhibited stronger biological activity, respectively. Furthermore, preliminary phytochemical tests were analyzed on total n-hex extracts in both species, which showed that the existence of common phytocomponents such as steroids play the major role to indicate the growth inhibition effect. GC-MS analysis of potent extract and its fractions in both potent species (Tables 4 and 5) showed various compounds. In the case of *E. caeruleum*, n-hexadecanoic acid, 7, 10 –pentadecadiynoic acid, methyl 6,9-octadecadiynoate in 40%, and 60% VLC fractions of n-hex extract were in high amounts. Moreover, hydrocarbons in n-hex extract were allocated as high amount of compounds. On the other hand, in the *E. thyrosoideum*, the main compounds in n-hex extract and 40% fraction were hydrocarbons (such as: hentriacontane) and fatty acids (7, 10-pentadecadiynoic acid), respectively. Presence of some steroids, terpenoids, stigmasterol methyl ether, campesterol, spathulenol, limonen-6-ol, pivalate, nerolidol-epoxyacetate, phytol, falcarinol, and eugenol even in low amounts, in the 40% VLC fraction of n-hex extract of *E. thyrosoideum* is considerable.

**Table 1:** List of plants, their collection site, extracted parts in the study and traditional use

**Plant species, family**	**Acquisition code number/Collection site**	**Extracted part**	**Traditional use of this plant or other species (Mozaffarian, 2013)**
*Anthriscus nemorosa, *Apiaceae	(Tbz-fph 1037) Namin	Aerial parts	Septic wounds, eczema
*Artemisia marschalliana, *Asteraceae	Arasbaran	Aerial parts	Malaria, fungal disease, fever
*Ecballium elaterium, *Cucurbitaceae	(Tbz-fph 648) Moghan	Seeds	Hypertension, constipation, tinea
*Eryngium billardieri, *Apiaceae	(Tbz-fph 1249) Maragheh	Aerial parts	anti-inflammatory, anti-fungal, anti-microbial
*Eryngium caeruleum, *Apiaceae	(Tbz-fph 1800) Namin	Aerial parts	Cough, epilepsy, anti-microbial
*Eryngium thyrsoideum, *Apiaceae	(Tbz-fph 1250) Maragheh	Aerial parts	anti-inflammatory, anti- microbial
*Lepidim vesicarium, *Brassicaceae	(Tbz-fph 1554) Moghan	Aerial parts	Anti-virus, anti-bacterial, diuretic

**Table 2:** Antimicrobial activity of plant extracts using agar disc diffusion method

**Plant**	**Extracts**	**Diameter of Inhibition zone (DIZ, mm)**						
		***S.a***	***S.e***	***B.s***	***L.m***	***E.c***	***S.t***	***C.a***
*A*. *nemorosa*	n-hex	-	++	++	+	-	-	++
	DCM	-	++	++	-	-	-	-
	MeOH	-	-	+	+	-	-	-
*A. marschalliana*	n-hex	-	++	++	+	-	-	-
	DCM	-	++	++	++	-	-	-
	MeOH	-	-	-	-	-	-	-
*E*. *elaterium*	n-hex	-	-	-	-	-	-	-
	DCM	-	-	+	-	-	-	-
	MeOH	-	-	-	-	-	-	-
*E*. *billardieri*	n-hex	-	+	+	+	-	-	-
	DCM	-	-	-	-	-	-	-
	MeOH	-	-	-	-	-	-	-
*E*. *caeruleum*	n-hex	+++	+++	++	++	-	-	-
	DCM	-	-	+	-	-	-	-
	MeOH	-	-	-	-	-	-	-
*E*. *thyrsoideum*	n-hex	-	+++	++	++	-	-	-
	DCM	-	++	+	-	-	-	-
	MeOH	-	++	-	-	-	-	-
*L*. *vesicarium*	n-hex	-	-	-	-	-	-	-
	DCM	++	+	+	++	-	-	++
	MeOH	-	-	-	-	-	-	-

+: DIZ = 7-9 mm; ++: DIZ = 10-14 mm; +++: DIZ > 15 mm; -: no activity; S.a: *Staphylococus aureus; *S.e: *Staphylococus epidermidis*; B.s: *Bacillus subtilis*; L.m: *Listeria monocytogenes*; E.c: *Escherichia coli*; S.t: *Salmonella typhi*; C.a: *Candida albicans.*

**Table 3:** Diameter of inhibition zone (DIZ, mm) and minimum inhibitory concentration (MIC, mg mL-1) of n-hex extracts of *E. caeruleum *and *E. thyrsoideum *and their potent VLC fractions against microorganisms

**Plant**	**n-hex extract and VLC fractions (EtOAC: n-hex)**	**DIZ (mm)**		**MIC (mg/mL)**	
		***S. aureus***	***S. epidermidis***	***S. aureus***	***S. epidermidis***
*E. caeruleum*	n-hex	18	19	6.25	0.781
	10:100	11	12	12.5	12.5
	20:80	13	12	6.25	6.25
	40:60	+	14	12.5	1.562
	60:40	18	20	6.25	1.562
	80:20	+	12	25	3.125
	EtOAc	+	12	25	3.125
*E. thyrsoideum*	n-hex	-	17	-	1.562
	10:100	-	-	-	-
	20:80	-	13	-	3.125
	40:60	-	21	-	0.39
	60:40	-	11	-	6.25
	80:20	-	12	-	6.25
	EtOAc	-	12	-	3.125

+: DIZ < 10 mm; -: No inhibition.

**Table 4:** Volatile components of n-hex extract, 40% and 60% VLC fractions of n-hex extract of *E. caeruleum.*

**NO.**	**n-hex**			**40% VLC fraction**			**60% VLC fraction**			**Identification method** ^b^
	**Compound** ^a^	**Area (%)**	**KI**	**Compound**	**Area (%)**	**KI**	**Compound**	**Area (%)**	**KI**	**GC-MS, KI**
1	1-Hexene	17.57	585	1-Hexene	14.6	585	3,4-Dimethylheptane	1.48	900	GC-MS, KI
2	Hexanoic acid	5.66	962	3,4-Dimethylheptane	3.2	900	Octanal	4.83	988	GC-MS, KI
3	Octanal	4.39	992	Dihydroactinidiolide	4.03	1473	Octanoic Acid	5.17	1165	GC-MS, KI
4	Caprylic acid	6.82	1165	(+) spathulenol	7.04	1578	Ethyloctynol	1.26	1240	GC-MS, KI
5	Lauraldehyde	1.64	1388	n-Hexadecanoic acid	20.52	1940	2-(3-Oxobutyl)cyclohexanone	0.77	1410	GC-MS, KI
6	1-Dodecanol	8.87	1457	7,10-Pentadecadiynoicacid	25.22	-	Azelaic Acid	1.08	1439	GC-MS, KI
7	Cyclododecane	2.61	1519	Methyl 6,9-octadecadiynoate	20.49	-	1-Tridecanol	2.49	1460	GC-MS, KI
8	Hexadecane	10.04	1600				1-Methyl-5-nitroimidazole	1.57	1534	GC-MS, KI
9	Ethanol, 2-(dodecyloxy)-	8.76	1702				2-(Dodecyloxy)ethanol	3.91	1698	GC-MS, KI
10	1-Tetradecanol	1.06	1718				NEOPHYTADIENE	1.91	1835	GC-MS, KI
11	Hexahydrofarnesyl acetone	0.83	1846				n-Hexadecanoic acid	20.44	1940	GC-MS, KI
12	n-Hexadecanoic acid	4.33	1940				(Cetyloxymethyl)oxirane	0.98	1973	GC-MS, KI
13	Ethyl hexadecanoate	5.43	1978				Hentriacontane	1.97	3100	GC-MS, KI
14	Tricosane	3.68	2300				Delta-Stearolactone	1.01	-	GC-MS, KI
15	Nonacosane	11.08	2900				7,10-Pentadecadiynoic acid	26	-	GC-MS, KI
16	Vinyl stearat	3.02	-				1-Phenyl-1-nonyne	3.99	-	GC-MS, KI
17	1,8-Cyclotetradecadiyne	1.68	-				1-Allyl-2-methylenecycloheptanol	6.92	-	GC-MS, KI
18							Bis (di-tert-butylbismutino)sulfurimide	2.36	-	GC-MS, KI
Total compounds		97.47			95.10			86.79		
Non terpenoid		96.64			88.06			86.79		
Fatty acids and derivatives		36.87			66.23			55.10		
Hydrocarbones		59.77			21.83			31.69		
Terpenoids		0.83			7.04			-		

^a^ **Compounds listed in order of elution from a DB-1 column.**

^b^ **Identification Method (KI = Kovats retention index according to authentic standard**

**Table 5:** Volatile components of n-hex extract and 40% VLC fraction of n-hex extract of* E. thyrsoideum*

**NO.**	**n-hex**			**40% VLC fraction**			**Identification method** ^b^
	**Compound** ^a^	**Area (%)**	**KI**	**Compound**	**Area (%)**	**KI**	
1	6-Methyltridecane	4.68	1300	2,4,6-Trimethyloctane	0.46	1100	GC-MS, KI
2	Hentriacontane	14.48	1312	(+)-Spathulenol	0.59	1577	GC-MS, KI
3	Eugenol	0.48	1345	Androstan-17-one, 3-ethyl-3-hydroxy, (5.alpha.)	1.38	1608	GC-MS, KI
4	1-Tridecanol	12.62	1457	Aromadendrene oxide-(2)	1.36	1638	GC-MS, KI
5	Hexadecane	21.72	1600	Longipinocarveol, trans-	3.31	1682	GC-MS, KI
6	5-Octadecene	4.44	1661	Limonen-6-ol, pivalate	0.63	1739	GC-MS, KI
7	2-(Dodecyloxy)ethanol	16.94	1699	Nerolidol epoxy acetate	0.74	1820	GC-MS, KI
8	Nor- pristan	2.59	1800	Neophytadiene	0.9	1839	GC-MS, KI
9	Neophytadiene	1.24	1836	Hexadecanoic acid	4.58	1940	GC-MS, KI
10	n-Hexadecanoic acid	3.88	1940	Falcarinol	0.65	2000	GC-MS, KI
11	Ethyl palmitate	0.64	1978	Phytol	1.26	2098	GC-MS, KI
12	Eicosane	1.83	2000	Lanost-8-en-3β-ol	11.17	2462	GC-MS, KI
13	7,10-Pentadecadiynoicacid	4.66	-	Campesterol	1.18	3131	GC-MS, KI
14	Butoxytriglycol	3.06	-	7,10-Pentadecadiynoic acid	62.95	-	GC-MS, KI
15	Stigmasterol methyl ether	3.07	-	5,5-Diethyl-4-methylene-1,2-dioxolan-3-one	0.96	-	GC-MS, KI
Total compounds		96.03			94.19		
Non terpenoid		95.55			75.13		
Fatty acids and derivatives		20.05			71.43		
Hydrocarbones		75.8			3.7		
Terpenoids		0.48			19.06		

^a^Compounds listed in order of elution from a DB-1 column.

^ b^Identification Method (KI = Kovats retention index according to authentic standard).

## Discussion

Microorganisms are considered as the mainstay and origin of multiple diseases. Germs have been identified as infectious agents in many diseases. Most commonly agents used as antimicrobial drugs have lost their effectiveness in therapeutic doses against microorganisms, and with increasing doses, the side effects of drugs increase and endanger human life, so this is a major concern of these days in the world (20). According to recent studies, naturally occurring compounds such as plants can be a good solution to this problem. In various scenarios, enormous interests in searching for new anti-microbial compounds from plants which can overcome to drug resistance have been actuated (21, 22). The present study was leaded to examine the *in-vitro* antimicrobial activity of some plants (twenty-four extracts of 8 species of plants from 4 families) used in traditional medicine of Iran against four gram positives, two gram negatives and a fungi to investigate their advantages compared with common antibiotics. The disk diffusion and modified broth micro-dilution methods were applied for determining the anti-microbial activity of different concentrations of extracts and fractions. Presence or absence of inhibition zone indicates the potency of various kinds of extracts. Five plants including *A. nemorosa, A. marschalliana*, *E. caeruleum, E. thyrsoideum* and* L. vesicarium* illustrated broad scale of anti-microbial activity. The same reports on anti-microbial effects of Iranian medicinal plants such as essential oil of the root of *Anthriscus nemorosa*, silver nanoparticles of *Artemisia marschalliana,* fruits of *Ecballium elaterium,* essential oil of aerial parts of *Eryngium caeruleum, *and ethanolic extracts of *Lepidium vesicarium, *were also evaluated, and demonstrated different results in comparison to our work with varying degrees of potency (23-31). In our study, the sensitivity tested microorganisms was, in decreasing order: *S. epidermidis, S. aureus, B. subtilis*, *L. monocytogenes*, *C. albicans*, *E. coli*, and *S. typhi. *As a whole, different extracts of the plants were effective on gram-positive bacteria and among these strains, *S. epidermidis* and *S. aureus* were more affected by several extracts. Furthermore, MeOH extract of *A*. *nemorosa* and n-hex extract of *L*. *vesicarium* were effective on *C*. *albicans*, as opposed to the rest of the extracts. None of the extracts had any effect on gram-negative bacteria. In the case of the tested bacteria, it has been mentioned that the possible cause of the difference in bacterial susceptibility is the presence of an outer membrane around the cell wall of the gram-negative bacteria that prevents the release of the extract through the lipopolysaccharide coating (32). In other words, differences between the ingredients of gram positive and gram negative cell walls were as a main cause for showing different susceptibility. Moreover, in the periplasmic space of gram negative species, there are enzymes degrading external molecules (33). Among the gram positive bacteria, *B. subtilis* has showed minimum sensitivity in comparison to other species, which may be due to its capability to form endospores as an enormous resistant material. All of the gram negative species were more resistant upon the samples. The n-hex extracts of *E*. *caeruleum* and *E*. *thyrsoideum* caused significant anti-microbial effects on *S. aureus* and *S. epidermidis*. In the case of *E. caeruleum* the inhibition activity against *S. aureus* and *S. epidermidis* was approximately the same. However, *S. epidermidis* in comparison to *S. aureus* was more affected by *E. thyrsoideum*. *S. aureus *for having broad range of distribution on normal body flora, showed resistance against many drugs in comparison to *S. epidermidis *(34). In order to further investigation and identify anti-microbial compounds, different fractions of potent extracts (n-hex) of two plants (*E. thyrsoideum* and *E. caeruleum*) were obtained by VLC method using different percentages of EtOAc and n-hex, and also their anti-microbial activity was measured against more sensitive strains. 60% and 40% VLC fractions in *E. caeruleum* and 40% fraction in *E. thyrsoideum* both against *S. epidermidis *exhibited significant effect, respectively. However, MIC amount of n-hex extract of *E. caeruleum* in comparison to VLC fractions is low; in the case of *E. thyrosoideum* this quantity is vice versa. Hence, it is concluded that VLC fractions of *E. caeruleum* showed their inhibitory effect synergistically, but in the *E. thyrosoideum* main antimicrobial compounds have been accumulated in 40% VLC fractions. In many other species of *Eryngium*, anti-microbial activities have been investigated, but their findings have been completely different with ours. Although, in different studies, various species of *Eryngium* inhibited gram positive strains (35, 36), in Bazzaz and *et al.,* research *E. billardieri* indicated a considerable activity against gram negative strain (*E. coli*). In our current assay, it did not show considerable growth inhibition effect on studied microbial strains (37). In spite of our finding (ineffectiveness of MeOH extract), in Marčetić *et al.*’s essay, MeOH extract of *E. palmatum* exerted potent effect on both gram positive and negative species (38). The differences in potency may be due to the different sensitivity of the test strains along with method of extraction. Hence, in order to determine the main differences, phytochemical analysis of n-hex extract, 40% and 60% VLC fractions of *E. caeruleum* as well as n-hex extract and 40% VLC fraction of *E. thyrsoideum* as potent agents were investigated by GC-MS analysis. Furthermore, preliminary phytochemical tests in both species showed that steroids play the major antimicrobial role. Our results in some extent are in consistent with the reports of the other researchers (36, 38 and 39). On the other hand, in n-hex extracts and 40%, 60% VLC fractions in potent species (*E*. *caeruleum* and *E. thyrosoideum*) fatty acids and derivatives, hydrocarbons and terpenoids were observed as the most common active compounds in a large part of volatile components, correspondingly. In the case of *E. thyrosoideum*, the main compounds were hydrocarbons and fatty acids. Furthermore, the presence of some steroids, terpenoids and potent anti-microbial agents like stigmasterol methyl ether, campesterol, spathulenol, limonen-6-ol, has caused the 40% VLC fraction showing considerable anti -microbial activity with minimum IC_50_. The anti-microbial effect of some above mentioned secondary metabolites have been proved by many literatures (40-46). Furthermore, different previous investigations have confirmed the anti -microbial activity of steroids and fatty acids (40, 44 and 47-52). It seems that steroids and fatty acids exert their anti-microbial potency by several mechanisms on cell membrane: Fatty acids create interim or perpetual hole on cell membrane and finally damage it for having amphipathic structure and detergent characteristic. Moreover, they can inhibit the activity of enzymes, ruin the nutrient absorption and finally can eradicate the existence of microbes by producing the free radicals (43, 47, 52 and 53). Furthermore, the findings of the previous studies speculated that the probable mechanism of anti-microbial action of terpenoids might result in the change of membrane penetrating and in permeation of intracellular agents (54, 55). Our anti-microbial screening findings affirm the traditional uses of some studied plants in different ailments containing infectious diseases.

## Conclusion

To sum up, among all the studied samples, n-hex extracts, 40%, 60% VLC fractions of *E*. *caeruleum* and 40% VLC fractions of *E. thyrosoideum *illustrated potent anti-microbial activities against *S. aureus* and *S. epidermidis *strains. Subsequently, their chemical analysis revealed potent anti-microbial agents. It is suggested to isolate the pure compounds of potent fractions and to elucidate their structure and to do anti-microbial tests on the purified compounds.
